# Recent advances in understanding dendritic cell development, classification, and phenotype

**DOI:** 10.12688/f1000research.14793.1

**Published:** 2018-09-26

**Authors:** Andreas Schlitzer, Wei Zhang, Mei Song, Xiaojing Ma

**Affiliations:** 1 LIMES-Institute, University of Bonn, Bonn, Germany; 2 Shanghai Institute of Cancer Research, Shanghai, China; 3 Department of Microbiology and Immunology, Weill Cornell Medicine, New York, NY, USA; 4 Sheng Yushou Center of Cell Biology and Immunology, School of Life Science and Biotechnology, Shanghai Jiao Tong University, Shanghai, China

**Keywords:** dendritic cell, development, differentiation, heterogeneity, tumor microinvironment, immunity, tolerance

## Abstract

Dendritic cells (DCs) play an essential role in the induction of adaptive immune responses against infectious agents and in the generation of tolerance to self-antigens. In this mini-review, we summarize new evidence suggesting that the tissue of residence significantly shapes the last developmental steps of DCs into locally adapted cellular entities, enabling them to perform tissue-specific tasks while maintaining the core DC properties. We also discuss recent advances that have highlighted DCs’ rather complex phenotypic and functional heterogeneity in the tumor microenvironment, based on their physical characteristics, such as activation status, maturity, and polarization, illustrating a key role for DCs in the induction of anti-tumor immunity.

## Dendritic cell development

Dendritic cells (DCs) have been progressively recognized as a separate hematopoietic lineage of myeloid cells, alongside granulocytes, macrophages, and monocytes. DC development is dependent on a cascade of bone marrow-resident hematopoietic stem cell (HSC)-derived precursor and progenitor cells. The first progenitor which has lost the potential to give rise to any other cell type except DCs is the common DC progenitor (CDP) ^1– 4^. The recent development of sophisticated methods ^5^ combined with the generation of refined mouse models for the conditional deletion of genes in DCs or the specific ablation of DCs or DC subsets ^6^ has helped to dissect the heterogeneity of DCs with respect to their developmental path, phenotype, localization, lineages, and function.

CDPs are characterized by high expression of Fms-like tyrosine kinase 3 (FLT3) alongside expression of colony-stimulating factor 1 receptor (CSF-1R) while retaining only an intermediate to low expression of the stem cell antigen kit. Furthermore, CDPs express high levels of interferon (IFN) regulatory factor 8 (IRF8), and their survival and continued development are critically dependent on this transcription factor ^7^. Additionally, CDPs express certain other stem cell-related transcripts such as cbfb and Runx 1 and 3 alongside Krüppel-like factor 4 (KLF4) and transcription factor 4 (TCF4) ^8^. CDPs gradually mature into pre-DCs ^9^. Pre-DCs can be identified by their high expression of FLT3 and CD11c and their intermediate to low expression of major histocompatibility complex II (MHC II). Interestingly, a subset of pre-DCs expresses the transcription factor Zbtb46 ^10^ and Siglec H ^11^. Further investigation into the heterogeneity within the pre-DC fraction leads to the identification of DC subset-committed pre-DCs. Subset-committed pre-DCs can be separated into four functionally and transcriptomically different subsets by using the surface markers Ly6c and Siglec H ^8^ or alternatively CD117 and Zbtb46 expression ^10^. Developmentally, four functionally and transcriptionally separate maturation stages of pre-DCs can be identified. Siglec H ^+^ Ly6c ^−^ pre-DCs are the developmentally earliest cells differentiating as CDP progeny. These cells still harbor the potential to give rise to conventional DCs (cDCs) that leave the bone marrow as precursors and plasmacytoid DCs (pDCs) that leave the bone marrow to go to the lymphoid organs and peripheral blood upon completing development ^12^. The consensus on the three major populations of murine DCs is that they are independently controlled by unique masters of transcriptional regulation ^13^ and bear these differential markers: conventional type 1 DCs (cDC1s) – CD8α ^+^ (lymphoid) and CD103 ^+^ (tissue), BATF3 and IRF8 dependent; conventional type 2 DCs (cDC2s) – CD11b ^+^ and CD172a ^+^, IRF4 dependent; and pDCs – IFNα-secreting, E2-2 dependent ^14^.

The potential to give rise to pDC progeny is lost upon maturing to the Siglec H ^+^ Ly6c ^+^ stage, as *in vitro* and *in vivo* differentiation assays clearly indicated only cDC progeny. Siglec H ^+^ Ly6c ^+^ pre-DCs differentiate under yet-unknown cues into two subsets: pre-cDC1, which specifically give rise to cDC1 in peripheral tissues, and pre-cDC2, which are dedicated precursors to cDC2. The molecular regulation of this subset-specific specification process is poorly understood. However, it seems that, for pre-cDC1 development, sustained and reinforced action of IRF8 and basic leucine zipper ATF-like transcription factor 3 (BATF3) is necessary, as revealed by sophisticated mutation analysis of the *BATF3* gene ^10^. Developmental specification of pre-cDC2, however, remains enigmatic. Pre-cDC1 and pre-cDC2 subsequently leave the bone marrow and seed peripheral organs giving rise to cDC1 and cDC2 under the influence of organ-specific microenvironmental cues, respectively.

In conclusion, cDC1 and cDC2 specification occurs at the pre-DC stage and is driven by subset-restricted progenitors locked into cDC1 or cDC2 fate. This knowledge now supports the assumption that a core DC subset transcriptome is established within the bone marrow environment under yet-unknown cues, allowing the formation of a cDC1 and cDC2 identity. Subsequently, within peripheral tissues, pre-cDC1 and pre-cDC2 fully develop into functionally mature cDC1 and cDC2, allowing the tissue to imprint an additional level of tissue-specific regulation on them to enable organ- and niche-specific functional adaptation.

Recently, a dedicated DC progenitor lineage has been identified in human bone marrow, peripheral blood, spleen, and cord blood. Reports by Breton *et al*. ^15^ and Lee *et al*. ^16^ first identified human CDPs in bone marrow and cord blood alongside a circulating pre-DC in peripheral blood. Subsequently, a study by See *et al*. was able to refine the definition of these precursor populations and show that DC subset-specific pre-DC subsets also exist in human peripheral blood as well as bone marrow and blood ^17^.

### Early imprinting of conventional dendritic cell identity

With the advent of single-cell transcriptomics and sophisticated genomic barcode-tracing strategies, it has become clear that the long-curated model of a stepwise hematopoietic development process is an oversimplification of myeloid hematopoiesis. Along these lines, studies using population-level barcode heterogeneity-tracing approaches revealed that a portion of HSCs contribute only to the DC repertoire and not to other repertoires, such as monocytes, or the lymphoid lineage, revealing that already at the HSC level a definitive fate decision can be made ^18^.

Furthermore, a single-cell transcriptomics approach, investigating the mouse CD117 ^+^ lineage marker-negative fraction, revealed that within the granulocyte macrophage progenitor (GMP) population, a population of transcriptomically pre-committed cells exist, showing the potential to give rise exclusively to DCs. This early DC progenitor upregulates hallmark transcriptional regulators of DCs, such as IRF8, inhibitor of DNA-binding 2 (ID2), and FLT3 alongside components of the MHC II antigen presentation pathway and gives rise preferentially to DCs, as shown in transplantation studies ^19^. However, the number of cells identified with a DC-specific transcriptomic program is small and most probably represents a physiological fallback mechanism or transient step toward the common CDP or a more committed DC progenitor. Additionally, substantial phenotypic overlap between the prior identified macrophage DC progenitor (MDP) ^20^ and this DC-committed GMP can be identified and should be investigated further. Taken together, evidence suggests that commitment to the DC lineage can be identified and maintained very early within the hematopoietic cascade and is most likely more prevalent than previously thought.

### Tissue factors influencing dendritic cell subset identity

Within peripheral tissues, several cues have been identified to contribute to the tissue-specific regulation and development of cDCs. The transcriptional target of Notch2, recombining binding protein suppressor of hairless (Rbpj), has been shown to be critical for the development of cDC2 in the spleen and has been speculated to be crucial to maintain splenic cDC2s within their specific tissue niche ^21^. Similarly, the G-protein-coupled receptor Epstein-Barr virus-induced gene 2 (EBI2), which recognizes 7α,25-dihydroxycholesterols, has been shown to regulate the positioning of cDC2 within the splenic microenvironment and its loss resulted in reduced numbers of cDC2, further strengthening the assumption that proper positioning within the spleen is crucial for cell survival and organ-adapted functionality ^22, 23^. Alongside these two niche-associated factors, V-Rel Avian reticuloendotheliosis viral oncogene homolog B (RELB), a component of the nuclear factor kappa B (NFκB) signaling network, exhibits crucial functions for splenic cDC2 development; however, the molecular regulation of this effect remains elusive ^24^.

Within peripheral organs such as the lung, granulocyte macrophage colony-stimulating factor (GM-CSF) has been explored as a tissue specification factor for cDCs. Indeed, Greter *et al.* were able to show that the maintenance and functional specialization of lung cDC1 are dependent on GM-CSF receptor signaling and, if perturbed, lead to loss of this subset and absence of T-cell responses toward particulate antigens, clearly identifying GM-CSF as a factor involved in tissue-specific imprinting of cDC development, maintenance, and function ^25^.

In the intestine, specifically in the small intestine, transforming growth factor-beta (TGF-β) was identified as the major driver for the tissue-specific differentiation of CD103 ^+^ CD11b ^+^ DCs (a subset of cDC2 in the intestinal microenvironment), a subset involved in the maintenance of intestinal T helper (Th) type 17 immunity and in the induction of intestinal Foxp3 ^+^ T cells, clearly showing the importance of such tissue-restricted functional imprinting on DC subsets ^26^.

Furthermore, within the skin, lung, and small intestine, a unique subset of CD103 ^−^ CD11b ^−^ DCs exists which depends on the transcription factor KLF4 and is crucial for the induction of protective Th2 immunity (for example, against parasites such as *Schistosoma mansoni*) ^27^. However, the exact mechanism of induction of KLF4 expression within this subset remains elusive. Interestingly, in mice devoid of KLF4 within their pre-DC compartment, pre-cDC1 and pre-cDC2 develop normally and also can be found within the affected tissues but are not able to develop further into their mature tissue-adapted progeny, indicating that KLF4 is upregulated in response to a local tissue-restricted factor.

Overall, ample evidence suggests that the tissue of residence significantly shapes the last steps of cDC development toward a fully tissue-adapted cDC, enabling it to perform tissue-specific tasks while maintaining core DC features such as antigen presentation and migration. This realization of a two-step differentiation process of cDCs will enable better utilization of cDCs in tissue-specific vaccination strategies in humans in health and in disease. However, Heidkamp *et al*. noted that phenotypic and transcriptional profiling of cDC and pDC subtypes in different human tissues derived from a large number of human individuals reveals that DC subpopulations in organs of the lymphohematopoietic system (spleen, thymus, and blood) are strongly defined by ontogeny rather than by signals from the microenvironment ^28^. In contrast, DC subsets derived from human lung or skin differed substantially, strongly arguing that DCs react toward modulatory signals from tissue microenvironments ^28^. In Table 1, we summarize the current understanding of human and murine DCs.

**Table 1.** Characteristics of human and mouse dendritic cells.

Classification	Main surface markers		Pathogen sensors		Major lineage TFs		Major cytokines	
	Murine	Human	Murine	Human	Murine	Human	Murine	Human
Plasmacytoid DC	CD45R CD45RA CD317 Siglec-H	CD123/IL-3R CD45RA CD303/CLEC4C CD304/BDCA-4 CD85κ/ILT3 CD85 *g*/ILT7 FCεR1 BTLA DR6/TNFRSF21 CD300A	TLR7 TLR9 TLR12 RLR STING	TLR7 TLR9 RLR STING	TCF4/E2-2 IRF7		IFN-α IFN-β IFN-λ IDO	IFN-α TNF IL-6 IDO
Myeloid cDC1	DEC205 CLEC9A XCR1	CD141/BDCA-3 CD13 CD33 CLEC9A CADM1/NECL2 BTLA XCR1	TLR2,3,4,9 11,12,13 STING	TLR1,3 TLR6,8 TLR10 STING	BATF3 IRF8 ID2 BLC6		TGFβ IL-12 IFN-λ	IFN-λ TNF-α IL-12 CXCL9 CXCL10
Myeloid cDC2	CD11b SIRPα	CD1c CD2 FCεR1 SIRPA CD11b CD11c CD1a * Langerin * CLEC10A/CD301a	All TLR except TLR3,11,12 RLR NLR STING	TLR2,4 TLR5,6 TLR8,9 RLR NLR STING	IRF4 KLF4 NOTCH2 RBPJ		IFN-α/β IL-1 IL-12 IL-23 TGFβ	IL-8 IL-1 IL-12 IL-23 TNF-α IL-10

DC, dendritic cell; TF, transcription factor; cDC, conventional dendritic cell; * inducible.

## Heterogeneity of dendritic cells in the tumor microenvironment

DCs are best known for their prominent role in the induction of adaptive immune responses against infectious agents and other types of “offensive” antigens, including tumor antigens ^29, 30^. However, recent advances have highlighted DCs’ rather complex phenotypic heterogeneity and functional plasticity, based on their attributes, such as activation status, maturity, and polarization in the tumor microenvironment (TME) ^31– 33^. TME is the cellular environment in which tumors coexist with the surrounding blood vessels, immune cells, fibroblasts, bone marrow-derived inflammatory cells, lymphocytes, and the extracellular matrix ^34, 35^. Tumors and TME interact constantly. Tumors can influence the microenvironment by releasing extracellular signals, promoting tumor angiogenesis, and inducing peripheral immune tolerance, while the components of TME can also affect the growth and evolution of cancerous cells ^36^.

Generally speaking, in TME, mature DCs are considered immune stimulatory whereas immature DCs are thought to be suppressive and tolerogenic. Tumor-infiltrating DCs (TIDCs) have distinguishable markers, such as CD11c ^high^/MHC II ^high^ CD11b ^+^ CD103 ^−^ PD-L1 ^+^ IL4Rα ^+^ DCs, found in lung cancer ^37^. TIDCs have been found in TME in many cancer types, such as breast, colorectal, lung, renal, head and neck, bladder, gastric, and ovarian ^38^. Their activities are varied and highly complex. Moreover, cancer cells and their secreted immunosuppressive factors can undermine tumor immunity and disrupt functional differentiation and activation of DCs through various schemes, which are strong focus areas in cancer immunology.

### cDC1, cDC2, and monocyte-derived dendritic cells

Analyses of murine and human cancers have shown that tumor-resident DCs consist mainly of three developmentally distinct subsets based on their expression of the CD64, MerTK, CD11b, XCR1, signal regulatory protein a (Sirpa), and CD103 surface markers: cDC1, cDC2, and monocyte-derived DCs (Mo-DCs) ^39^. Mo-DCs differentiate from Ly6C ^+^ or CD14 ^hi^ monocytes in mice and humans, respectively ^40^. Tumor-resident Mo-DCs are characterized by their high expression of CD11b, CD64, and MerTK and are predominantly considered suppressors of anti-tumor immunity. Mouse cDCs can be classified into two functionally distinct lineages: the XCR1 ^+^ IRF8 ^+^ cDC1 lineage and the CD11b ^+^ IRF4 ^+^ cDC2 lineage. Siglec-H and Ly6C were identified as lineage markers that distinguished pre-DC subpopulations committed to the cDC1 lineage (Siglec-H ^−^ Ly6C ^−^ pre-DCs) or cDC2 lineage (Siglec-H ^−^ Ly6C ^+^ pre-DCs) ^8, 10^. cDC1s are also characterized by their high expression of XCR1 and have been reported to play predominantly an anti-tumor role. The cDC2s, in contrast, are characterized mainly by their high expression of CD11b and Sirpa and have been implicated in both anti- and pro-tumor mechanisms ^41^.

Mo-DCs are adept at tumor antigen uptake but lack strong T-cell stimulatory capacity because of nitric oxide-mediated immunosuppression and poor ability to migrate to tumor-draining lymph nodes ^39^. Flies *et al*. observed that CD11c ^+^ CD11b ^−^ CD103 ^+^ cDC1s were absent in the peritoneal cavity of healthy mice but comprise up to 40% of DCs in ovarian tumor-bearing mice and retained T-cell stimulatory capacity in advanced disease ^42^. Monocytes exposed to the appropriate conditions such as treatment with the immunostimulatory agents monosodium urate crystals and *Mycobacterium smegmatis* can become Mo-DCs and powerful activators of tumor-specific CD8 ^+^ T cells and anti-tumor immunity ^43, 44^. Among CD11c ^+^ CD11b ^+^ cDC2s, Lair-1 expression further distinguishes stimulatory and immunoregulatory DC subsets, which are also enriched in TME. Interestingly, programmed death-ligand 1 (PD-L1) is expressed by Lair-1( ^hi^) immunoregulatory DCs and may contribute to local tumor antigen-specific T-cell dysfunction ^42^. Like Mo-DCs, cDC2s were found to suppress cytotoxic T lymphocyte (CTL) function in tumor-bearing mice via L-arginine metabolism, among other potential modes of action ^45^, which is consistent with a previous finding that increased breakdown of the amino acids arginine and tryptophan in tumor-associated DCs negatively impacts T-cell effector function ^46^.

Using an *in vitro* culture model that produces human Mo-DCs and monocyte-derived macrophages (Mo-macrophages) closely resembling those found *in vivo* in ascites, Goudot *et al*. showed that the transcription factors IRF4 and MAFB were critical regulators of monocyte differentiation into Mo-DCs and Mo-macrophages, respectively ^47^. Furthermore, activation of the aryl hydrocarbon receptor (AHR) promoted Mo-DC differentiation through the induction of positive regulatory domain zinc finger protein 1 (BLIMP-1) while impairing differentiation into Mo-macrophages ^47^, demonstrating a critical role of AHR as a molecular switch for monocyte fate specification in response to TME-derived signals. These findings were further supported by Sander *et al*. ^48^, who demonstrated that *in vitro* generated Mo-DCs resemble monocyte-derived antigen-presenting cells (APCs) found in ovarian cancer-associated ascites ^49^.

### Plasmacytoid dendritic cells

pDCs are found in small numbers throughout the periphery and are recognized by their expression of B220, Ly6C, and PDCA.1 in mice and CD123, CD303/BDCA2, and CD304/BDCA4 in humans. Expression of SiglecH and Ly6D defined pDC lineage commitment along the lymphoid branch ^50^. pDCs selectively express Toll-like receptor 7 (TLR7) and TLR9, and their most important function is thought to be producing significant quantities of type 1 IFN in response to single-stranded viral RNA and DNA ^51^. pDCs also have the potential to act as APCs, as they express MHC II and co-stimulatory molecules; however, the ability of pDCs to phagocytose dead cells and present cell-associated antigen has not been clearly established nor has their ability to cross-present exogenous antigen on MHC class I ^12^. In human blood, single-cell RNA-sequencing analysis of blood DCs coupled with functional characterization has indicated that human pre-DCs contaminated the traditionally defined pDC gate and that this contamination is likely responsible for the previous misrepresentation of pDCs’ “T cell-activating” property ^52^. In tumors, the presence of pDCs seems to correlate with poor prognosis in both breast and ovarian cancers ^53, 54^, but pDCs can also act as therapeutic targets to elicit IFN-α release and antigen presentation by cDCs ^55, 56^. In mouse models of breast cancer, Wu *et al.* showed that activated pDCs can directly kill tumor cells through tumor necrosis factor-related apoptosis-inducing ligand (TRAIL) and granzyme B ^57^. Furthermore, pDCs initiated the sequential activation of natural killer cells and CD8 ^+^ T cells, which also contributed to inhibition of tumor growth ^57^.

### Inflammatory dendritic cells

New evidence suggests that tumors can convert TIDCs into immunosuppressive regulatory cells. A population of inflammatory DCs (inf-DCs) with a suppressive phenotype was described in the TME of different transplantable and autochthonous models of ovarian cancer ^58^. Inf-DCs originate from circulating Ly6C ^high^ monocytes as a consequence of inflammation, cancer, or infection ^59– 62^ and are generally absent under steady-state conditions. In mice, inf-DCs are identified as MHC II ^+^ CD11b ^+^ CD11c ^+^ F4/80 ^+^ Ly6c ^+^ and express CD206, CD115/M-CSFR, Mac-3/CD107b, FcεRI, and CD64 as well as the transcription factor zinc finger and BTB domain-containing 46 (Zbtb46). FcεRI appears to be a useful marker to distinguish inf-DCs from cDCs and macrophages. Several studies have shown that inf-DCs can activate antigen-specific CD4 ^+^ T-cell responses *ex vivo*. Inf-DCs can also cross-present exogenous antigens in different models, including Lewis lung carcinoma, HSV-1 reactivation, experimental autoimmune encephalomyelitis, and allograft rejection models ^63, 64^.

In conclusion, DCs of a heterologous nature are frequently recruited to tumor sites by specific tumor-derived and stroma-derived factors, which may impair DC maturation, differentiation, and function in TME, resulting in the deficient formation of anti-tumor immune response or development of DC-mediated tolerance and immune suppression ^65, 66^. These factors include, but are not limited to, growth factors such as vascular endothelium growth factor (VEGF), TGF-β, and growth/differentiation factor 15 (GDF-15) ^67– 69^; cytokines such as interleukin-6 (IL-6), IL-10, CSF1, and receptor activator of nuclear factor kappa-Β ligand (RANKL) ^70– 76^; and chemokines such as CCL2, monocyte inhibitory protein-3a (MIP-3a), stem cell factor-1 (SDF-1), mucin 1 (MUC1) ^77– 80^, and others such as prostaglandin E _2_ (PGE _2_) ^81, 82^ and PD-L1 ^31^. The tumor-derived immunoevasive and suppressive mechanisms constitute a major obstacle to the generation of effective anti-tumor immunity. Therefore, understanding the intercellular and intracellular circuits that modulate the immunogenic and tolerogenic phenotype of DCs in cancer may provide crucial insights for developing adjuvant treatments to alleviate immunosuppression in the TME and improve the clinical efficacies of cancer vaccines and immunotherapies.

## Abbreviations

AHR, aryl hydrocarbon receptor; APC, antigen-presenting cell; BATF3, basic leucine zipper ATF-like transcription factor 3; cDC, conventional dendritic cell; CDP, common dendritic cell progenitor; CSF, colony-stimulating factor; DC, dendritic cell; FLT3, Fms-like tyrosine kinase 3; GM-CSF, granulocyte macrophage colony-stimulating factor; GMP, granulocyte macrophage progenitor; HSC, hematopoietic stem cell; IFN, interferon; inf-DC, inflammatory dendritic cell; IL, interleukin; IRF, interferon regulatory factor 8; KLF4, Krüppel-like factor 4; M-CSFR, macrophage colony-stimulating factor receptor; MHC, major histocompatibility complex; Mo-DC, monocyte-derived dendritic cell; Mo-macrophage, monocyte-derived macrophage; pDC, plasmacytoid dendritic cell; PD-L1, programmed death-ligand 1; Sirpa, signal regulatory protein a; TGF-β, transforming growth factor-beta; Th, T helper; TIDC, tumor-infiltrating dendritic cell; TLR, Toll-like receptor; TME, tumor microenvironment
